# Endoreduplication is not involved in bundle-sheath formation in the C_4_ species *Cleome gynandra*

**DOI:** 10.1093/jxb/ert350

**Published:** 2013-11-12

**Authors:** Sylvain Aubry, Jana Kneřová, Julian M. Hibberd

**Affiliations:** Department of Plant Sciences, Downing Street, University of Cambridge, Cambridge CB2 3EA, UK

**Keywords:** Bundle sheath, C4 photosynthesis, *Cleome gynandra*, endoreduplication.

## Abstract

There is currently significant interest in engineering the two-celled C_4_ photosynthesis pathway into crops such as rice in order to increase yield. This will require alterations to the biochemistry of photosynthesis in both mesophyll (M) and bundle-sheath (BS) cells, but also alterations to leaf anatomy. For example, the BS of C_4_ species is enlarged compared with that in C_3_ species. Because cell and nucleus size are often correlated, this study investigated whether nuclear endoreduplication is associated with increased differentiation and expansion of BS cells. Nuclei in the BS of C_4_ *Cleome gynandra* were tagged with green fluorescent protein. Confocal laser-scanning microscopy and flow cytometry of isolated nuclei were used to quantify size and DNA content in BS cells. The results showed a significant endoreduplication in BS cells of *C. gynandra* but not in additional C_4_ lineages from both the monocotyledonous and dicotyledenous plants. Furthermore, in the C_3_ species *Arabidopsis thaliana*, BS cells undergo endoreduplication. Due to this significant endoreduplication in the small BS cells of C_3_ *A. thaliana*, it was concluded that endoreduplication of BS nuclei in C_4_ plants is not linked to expansion and differentiation of BS cells, and therefore that alternative strategies to increase this compartment need to be sought in order to engineer C_4_ traits into C_3_ crops such as rice.

## Introduction

From about 30 million years ago, plants using C_4_ photosynthesis evolved, and they now populate more than 60 independent lineages of angiosperms (Christin *et al.*, 2013). The C_4_ pathway reduces rates of photorespiration and therefore allows greater photosynthetic efficiency, primarily in open areas of the tropics and subtropics (Sage *et al.*, 2011). Although C_4_ species only represent around 3% of angiosperms (Sage *et al.*, 2011), it is estimated that they contribute to about 30% of net terrestrial primary productivity (Osborne and Beerling, 2006).

The C_4_ pathway involves the reactions of photosynthesis being divided between two compartments in the leaf, and this leads to CO_2_ being concentrated in bundle-sheath (BS) chloroplasts in full C_4_ plants that contain the primary carboxylase Ribulose Bisphosphate Carboxylase Oxygenase (RuBisCO). In all cases, carbonic anhydrase and phosphon*enol*pyruvate carboxylase convert CO_2_ into oxaloacetate in one compartment. The subsequent reduction or transamination of oxaloacetate to organic four-carbon acids such as malate and aspartate generate high concentrations of these metabolites, and this drives their diffusion into the second compartment. Here, one or more C_4_ acid decarboxylase releases high concentrations of CO_2_ around RuBisCO. In species that use the classic two-celled pathway, the high flux of metabolites between mesophyll (M) and BS cells is dependent on close contacts between these cell types, and typically this results in a stylized arrangement of each vein being surrounded by a ring of BS cells, which in turn is inside a ring of M cells, resulting in so-called Kranz anatomy (Hatch and Slack, 1966). The BS cells of C_4_ plants also contain many chloroplasts to increase the compartment volume containing RuBisCO after it is released by the C_4_ acid decarboxylases (Hattersley and Browning, 1981).

The C_4_ cycle is most often achieved by compartmenting photosynthesis between two cell types within the leaf, but it can occur within individual cells (Chuong *et al.*, 2006; Lara *et al.*, 2006). In species that use the two-celled C_4_ pathway, there is considerable variation in the exact cells within the leaf that fulfil the initial phosphon*enol*pyruvate carboxylase-dependent carboxylation step and the subsequent decarboxylation and refixation by RuBisCO (Dengler *et al.*, 1994). For example, there are at least 25 forms of Kranz anatomy (Edwards and Voznesenskaya, 2011) with four subtypes in the eudicots (Muhaidat *et al.*, 2007) and the most common arrangement being the atriplicoid subtype (Muhaidat *et al.*, 2007). The stylized pathway is often described as initial fixation in the M followed by decarboxylation in the BS, but in all cases, while in C_3_ species M volume is larger than that of the BS, the converse is true in C_4_ plants. This increase in volume of the BS in C_4_ leaves can be caused by it containing either more and/or larger cells (Muhaidat *et al.*, 2007; McKown and Dengler, 2009). As the increased productivity of C_4_ plants has led to the proposal that characteristics of C_4_ photosynthesis should be engineered into C_3_ crops such as rice to increase yield (Matsuoka *et al.*, 2001; Hibberd *et al.*, 2008), we need to understand the genetic basis underlying the expansion, differentiation, and specialization of the BS in C_4_ species.

In eukaryotes, there is often a positive correlation between cell size and nuclear DNA content (Sugimoto-Shirasu and Roberts, 2003; Lee *et al.*, 2009). Polyploid nuclei can occur as a result of a process called endoreduplication where chromosomal DNA replication is not followed by mitotic cell division. Endoreduplication is therefore a result of the canonical cell cycle G_1_-S-G_2_-M missing a mitotic cell division, and, as a result, this leads to an increase in nuclear DNA content (De Veylder *et al.*, 2007). In diploid organisms, endoreduplication can occur repeatedly during the lifetime of a cell, giving rise to multiple copies of the nuclear genome, ranging from 2C (the diploid state) up to 32–64C. In *Arabidopsis thaliana*, DNA content correlates with leaf age, and endoreduplication occurs once cells shift from proliferation to maturation (Beemster *et al.*, 2005). In *A. thaliana*, cell division is arrested along a gradient from the tip to the base of the leaf during secondary morphogenesis (Donnelly *et al.*, 1999). The physiological relevance of this gradual increase in nuclear DNA content is still a matter of discussion but can be induced during stress (Lee *et al.*, 2009). It can also be found in tissues such as the maize endosperm (Schweizer *et al.*, 1995) and hypocotyls of *A. thaliana* (Gendreau *et al.*, 1998), and is common in trichomes (Kasili *et al.*, 2010).

The correlation between nuclear ploidy state and cell size as well as organelle number has been reported in numerous plants (Kondorosi *et al.*, 2000; Sugimoto-Shirasu and Roberts, 2003; De Veylder *et al.*, 2011). For example, in floral apices of *Datura stramonium*, there is a direct correlation between ploidy, nucleus volume, and cell size, with larger cells having more DNA (Sugimoto-Shirasu and Roberts, 2003). In *A. thaliana*, ploidy levels of epidermal cells and trichomes show a positive correlation between DNA content and cell size (Melaragno *et al.*, 1993; Hulskamp *et al.*, 1999). While the direct link between ploidy and cell size is not always clear (Marks, 1997; John and Qi, 2008; Dissmeyer *et al.*, 2009), evidence that endoreduplication and cell enlargement are genetically linked is provided by analysis of *Medicago sativa* lines in which antisense repression of CCS52, which acts as a negative regulator of mitosis, led to both reduced ploidy and cell size (Cebolla *et al.*, 1999). If a C_4_ crop such as rice is to be engineered to use C_4_ photosynthesis, the BS will need to be increased in size (Sage and Zhu, 2011). To inform the C_4_ rice engineering effort, we therefore tested the hypothesis that expansion of the BS is associated with nuclear endoreduplication in this compartment. We demonstrate that the nuclei of BS cells of *Cleome gynandra* undergo endoreduplication, but that in other C_4_ species from independent C_4_ lineages within the angiosperms, this was not evident. Furthermore, we report that the endoreduplication in BS cells also occurred in C_3_ *A. thaliana*. We therefore infer that, to engineer C_4_ rice, alternate strategies will be needed to increase BS size.

## Materials and methods

### Cloning and production of transgenic lines

The vectors used were derived from the INTACT systems (Deal and Henikoff, 2010), which target green fluorescent protein (GFP) to the nuclear envelope. The *FtGLDPA* (Wiludda *et al.*, 2012) promoter was amplified as a 1200bp fragment with *Xma*I and *Nhe*I restriction sites included in the primers *Xma*I-*Ft*GLDp-F 5′-CACCCCCGGGAAGCTTTACTCCTCTCAAC-3′ and *Nhe*I-*Ft*GLDp-R 5′-TTTGCTAGCTAGTGTAAGATGGGGTCTA-3′. This allowed the fragment to be cloned into the GL2p:NTF vector (Deal and Henikoff, 2010) at the *Xma*I and *Nhe*I sites. The promoter+NTF region was then amplified by PCR and cloned into pENTR/D-TOPO with a CACC overhang on the 5′ primer to orientate the cloning. Clones containing the promoter and the NTF were sequenced and subsequently inserted into the binary vector pGWB1 (Nakagawa *et al.*, 2007) by Gateway LR recombination. *Agrobacterium tumefaciens* LBA4404 was transformed with this construct and selected on both kanamycin and streptomycin. *C. gynandra* callus was transformed as described previously (Newell *et al.*, 2010). Briefly, 10-d-old hypocotyls and cotyledons explants were sectioned and dipped in liquid MS medium supplemented with vitamins and 0.2mM acetosyringone (pH 5.5) containing the resuspended *A. tumefaciens* culture. After 30min, explants were transferred for 2 d to co-culture plates (MS medium at 1/10 normal concentration, 30g l^−1^ of sucrose, 1mg l^−1^ of benzylaminopurine, 0.1mg l^−1^ of naphthalene acetic acid, 8g l^−1^ of agar, pH 5.5, covered with 1ml MSO liquid), and finally placed on regeneration medium with antibiotics for 3 weeks. Explants were then grafted onto wild-type root stocks to allow seed production. Three independent T_1_ transgenic lines were used in this study.

### Plant material and microscopy

*C. gynandra* (C_4_) and *A. thaliana* (C_3_) were grown in soil under long-day conditions (16h light, 8h night) in a cabinet set at 150 µmol photons m^–2^ s^–1^ and a temperature of 23 °C during the day and 20 °C during the night. To assess endoreduplication, leaves were harvested from 5 to 30 d after planting and analysed by flow cytometry. For analysis of cell and nuclei size in M and BS cells, *Atriplex rosea* (Amaranthaceae), *Flaveria trinervia* (Asteraceae). *Zea mays* (Poaceae), and *Setaria viridis* (Poaceae) were grown for 5 weeks, and mature fully expanded leaf sections were harvested. Fresh leaves were embedded in 5% agarose, and 50 µm sections were cut with a vibratome. Sections were stained with propidium iodide or 4′-6-diamidino-phenylindole (DAPI) and visualized using a 488nm laser of a Leica TCS SP5 confocal microscope. GFP and chlorophyll were excited with a 488nm laser and emission was detected at 495–530 and 650–670nm, respectively. Chlorophyll and DNA were detected in different channels and are presented overlaying each other. Areas of cells and nuclei were calculated using Photoshop CS6 from at least five independent biological replicates and statistically significant differences (*P*<0.05) were determined using a one-tailed *t*-test.

### Flow cytometry

DNA content was determined as described previously (Zhang *et al.*, 2005; Dolezel *et al.*, 2007) (see Supplementary Fig. S1, at *JXB* online). Briefly, 20mg of fresh leaves was chopped with a razor blade for 30 s in 1ml of 45mM MgCl_2_, 20mM MOPS, 30mM sodium citrate (pH 7) and 0.1% Triton X-100. The homogenate was filtered through a 40 µm nylon mesh and nuclei were stained with 2 µg ml^–1^ of DAPI. Flow cytometry was performed on a Dako Cyan cytometer using 365 and 488nm lasers. GFP fluorescence, excited by the 488nm beam, was diverted using the dichroic filter 545 DLP and detected using the FL1 channel (530/540nm). DAPI fluorescence, excited by the 365nm beam, was routed directly on the FL7 channel (450/450nm). A total of 200 000 events were counted at a rate of 200 nuclei s^–1^ and at least 20 000 gated events were used for counting. Biparametric histograms of log(DAPI) versus log(GFP) signals were used to select DAPI-positive particles (nuclei), and GFP-positive nuclei were sorted according to their ploidy levels (see Supplementary Fig. S3, at *JXB* online, showing a representative trace of BS cells ploidy in *A. thaliana*). Means ±SEM were calculated after sampling three biological replicates and conducting the time-course experiment twice.

## Results

### BS nuclei are larger than those in mesophyll cells of *C.* *gynandra*

**Fig. 1.**
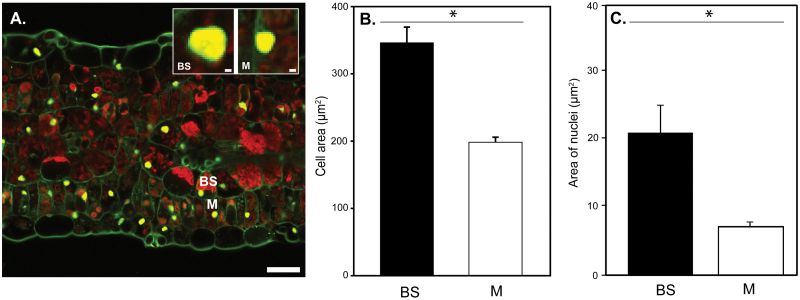
Nuclei of BS cells are larger than those of M cells of *C. gynandra.* (A) Representative transverse section of a mature *C. gynandra* C_4_ leaf stained with propidium iodide and imaged with CLSM. Nuclei and cell walls are yellow while chlorophyll fluorescence is red. (B, C) Quantitation of BS and M cell area (B) and area of nuclei (C) of *C. gynandra*. Data and images are representative of five independent leaves. Bars, 25 µm (main picture); 1 µm (insets). Asterisks represent statistically significant differences between BS and M (*P*<0.05).

Confocal laser-scanning microscopy (CLSM) showed that, in mature leaves of *C. gynandra*, BS cells were 1.8 times larger than M cells (Fig. 1A, B). Propidium iodide staining of nuclei established that those in the BS were around three times larger than those in M cells (Fig. 1A, C). It was also apparent that nuclei in BS cells were often located between the centripetally arranged chloroplasts and the vacuole, whereas nuclei in the M were scattered randomly within each cell (Fig. 1A).

### Endoreduplication in BS nuclei occurs in C_3_ and C_4_ species

**Fig. 2.**
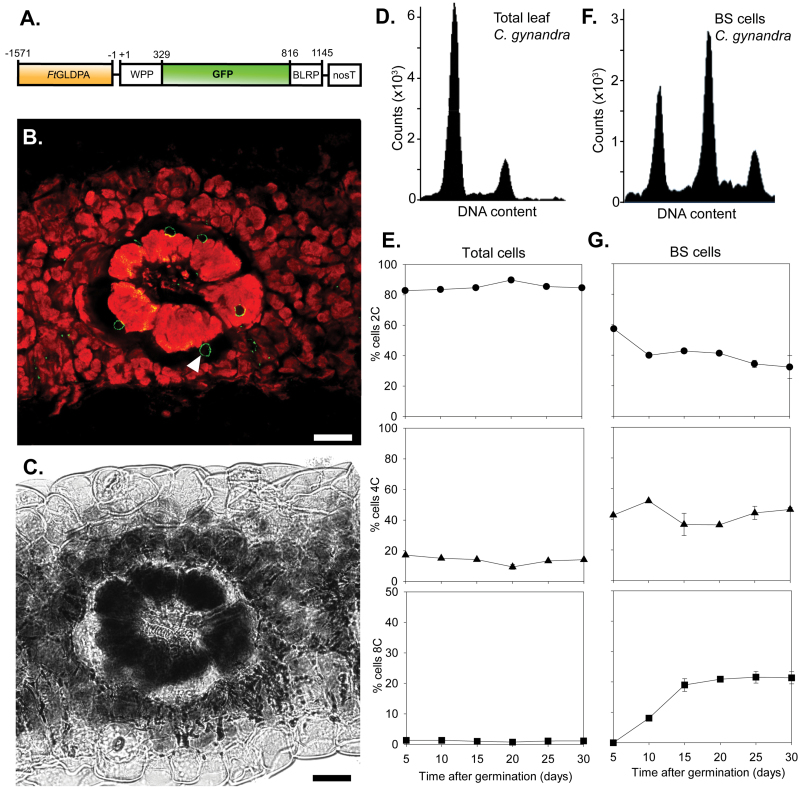
BS nuclei of *C. gynandra* endoreduplicate during leaf maturation. (A) Schematic of the construct targeting GFP to BS cells. GFP was translationally fused both to the WPP motif allowing nuclear localization and to a biotin ligase peptide. The promoter from the *F. trinervia* glycine decarboxylase promoter (Wiludda *et al.* 2011) was used to drive expression in BS cells of *C. gynandra*. *FtGLDPA*, *F. trinervia* glycine decarboxylase subunit P promoter; WPP, nuclear envelope targeting peptide; BLRP, biotin ligase; nosT, *nos* terminator. (B, C) Representative transverse sections of a *C. gynandra* leaf expressing the BS tagging construct. A transverse section after CLSM with chlorophyll (red) and GFP (green) signals overlaid is shown in (B). BS nuclei in *C. gynandra* were often seen between the vacuole and the chloroplasts (white arrowhead). A bright field image of the same section is shown in (C). (D) Representative flow cytometry trace showing the DNA content of mature *C. gynandra* leaves. (E) Ploidy levels as leaves mature. Total leaf DNA content was analysed after flow cytometry of DAPI-stained nuclei. (F) Representative flow cytometry trace showing the DNA content of BS cells in mature *C. gynandra* leaves. (G) Ploidy as *C. gynandra* leaves matured. Bars, 25 µm. Data are shown as means ±1 SEM.

To investigate whether the increase in BS size was associated with endoreduplication, we generated lines in which nuclei from BS cells contained GFP. Nuclei in BS cells were labelled using the BS-specific *Flaveria trinervia* glycine decarboxylase (*FtGDLPA*) promoter (Wiludda *et al.*, 2012) to drive expression of a translational fusion between GFP and the WPP domain of the Ran GTPase activating protein (RanGAP1), which is targeted to the nuclear envelope (Deal and Henikoff, 2010, 2011) (Fig. 2A). CLSM confirmed that this construct led to specific expression in BS cells of *C. gynandra* (Fig. 2B, C). When nuclei from all cell types of the leaf were assessed, 80% were 2C, with only 18 and 2% being 4C and 8C, respectively (Fig. 2D, E; Supplementary Fig. S2 at *JXB* online). This was maintained from 5 to 30 d after germination as the leaves matured (Fig. 2E). In contrast, when fluorescently activated cell sorting was used to separate GFP-labelled BS nuclei from leaves, this established that the proportion of 2C and 8C nuclei declined and increased, respectively, as leaves matured (Fig. 2F, G).

**Fig. 3.**
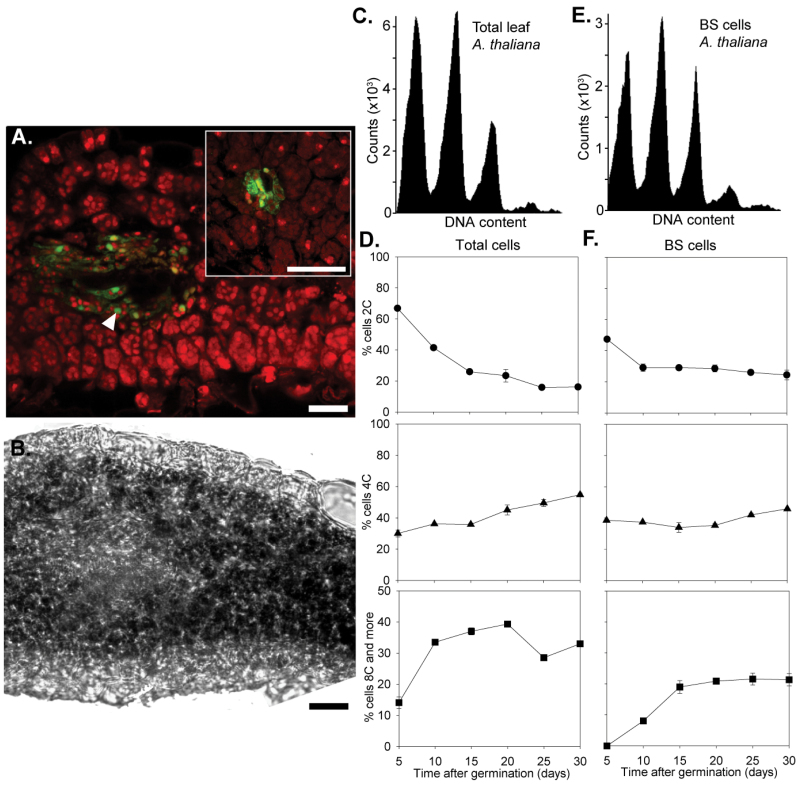
BS nuclei of *A. thaliana* endoreplicate during maturation. (A) Representative transverse section observed after CLSM of *A. thaliana* leaves. Chlorophyll (red) and GFP (green) signals are overlaid, the white arrowhead points at a BS nucleus marked with GFP. (B) Bright-field image of the same section. (C) Total leaf DNA content analysed by flow cytometry of DAPI-stained nuclei. (D) Change in ploidy as leaves age. (E) Representative flow cytometry trace showing DNA content of BS cells in mature *A. thaliana* leaves. (F) Change in ploidy of BS as *A. thaliana* leaves age. Bars, 25 µm. Results are shown as means ±1 SEM.

The *FtGDLPA* promoter directs BS specificity in *A. thaliana* (Wiludda *et al.*, 2012), and so to investigate whether the increased DNA content of BS nuclei is an ancestral characteristic, we transformed the same *FtGDLPA::GFP::RanGAP1* construct into C_3_ *A. thaliana* (Fig. 3A, B). When nuclei from all cells of *A. thaliana* leaves were separated and DNA content assessed, this showed that there was a gradual reduction in 2C content as leaves matured and a consequent increase in 4C and 8C content (Fig. 3C, D). This trend was also evident in nuclei isolated from BS cells (Fig. 3E, F) and interestingly the increase in 8C nuclei in BS cells was very similar in C_3_ *A. thaliana* and C_4_ *C. gynandra* (Figs 2G and 3F).

**Fig. 4.**
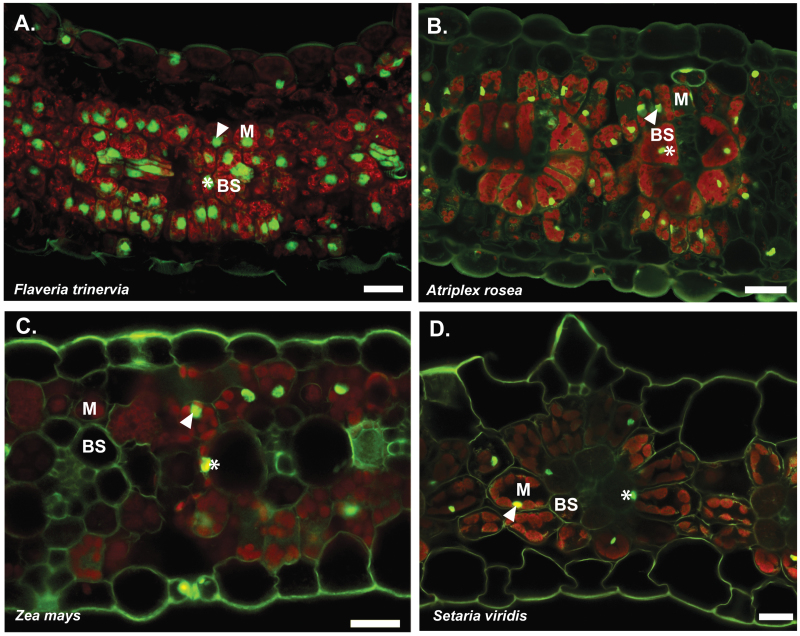
BS and M nuclei from four species with independent C_4_ origins. Representative transverse sections of leaves stained with propidium iodide and imaged with CLSM. Nuclei and cell walls are green, while chlorophyll fluorescence is red. Results are shown for *F. trinervia (*A), *A. rosea* (B), *Z. mays* (C), and *S viridis* (D). Nuclei in M and BS cells are annotated with arrowheads and asterisks, respectively. Five biological replicates were assessed. Bars, 25 µm.

To investigate the extent to which other C_4_ plants possess larger nuclei in BS compared with M cells, we analysed two additional C_4_ dicotyledons, *Flaveria trinervia* and *Atriplex rosea*, and also two monocotyledonous species, *Zea mays* (maize) and *Setaria viridis*. In contrast to *C. gynandra*, nuclei in BS and M cells were of a similar size in all four species (Fig. 4 and Table 1). This was probably caused by endoreduplication in both cell types. Furthermore, the size of BS cells from *F. trinervia*, maize and *S. viridis* were not significantly larger than M cells (Table 1). Together, these data indicated that expansion of the BS compartment in these C_4_ species was due to a larger number, rather than larger size, of individual cells.

**Table 1.** Quantitative analysis of cell and nucleus area in A. thaliana and four C_4_ speciesData are derived from at least five biological replicates and shown as mean ±1 SEM. Statistically significant differences are depicted by asterisks (*P<0.05; **P <0.001).

Species	C_4_ subtype	Cell area (µm^2^)		Nucleus area (µm^2^)	
		BS	M	BS	M
*A. thaliana*	–	194.5±15.9**	319.4±17.2**	6.2±0.3	6.3±0.2
*F. trinervia*	NADP-ME	130.5±7.5	127.4±8.5	28.4±1.4	32.1±1.5
*A. rosea*	NAD-ME	314.4±19.7*	244.0±12.4*	13.5±1	14.4±1.2
*Z. mays*	NADP-ME	246.0±46.6	356.2±23.2	31.6±3.4	27.9±1.1
*S. viridis*	NADP-ME	213.4±14.3**	336.5±22.3**	9.3±0.7	8.3±0.3

**Fig. 5.**
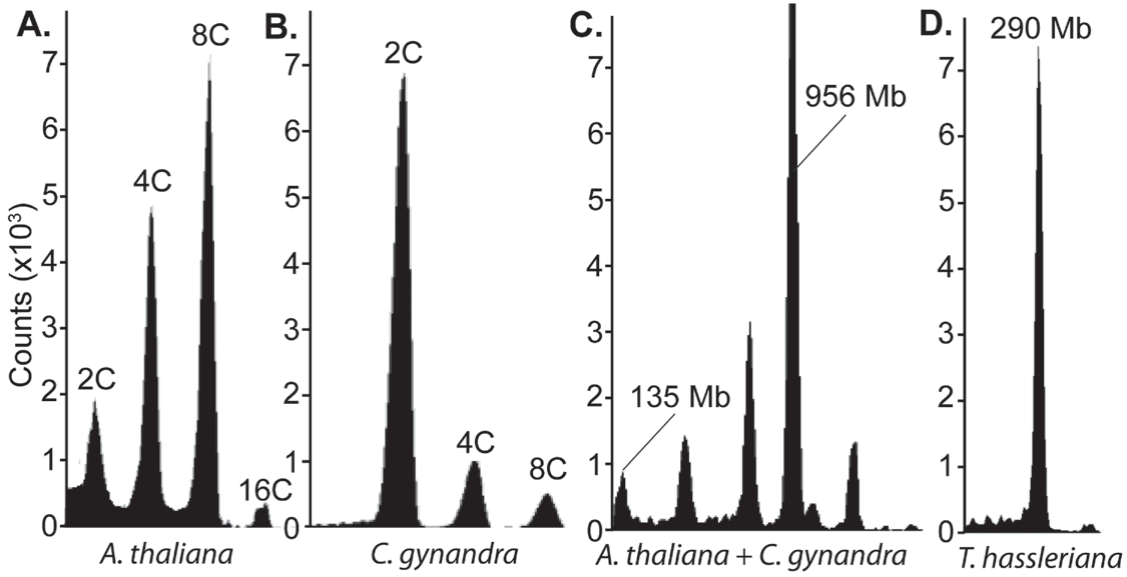
Genome size of *C. gynandra*. Flow cytometric analysis of DAPI-stained *A. thaliana* and *C. gynandra* wild-type leaves were performed either separately (A, B) or together (C). Log-scaled DAPI signals are plotted against the event counts. Based on the size of the *A. thaliana* genome (135Mb), we inferred a genome size for *C. gynandra* of approximately 956Mb. (D) Flow cytometric analysis of a *Tarenaya hassleriana* mature leaf, a closely related C_3_ of *C. gynandra*.

We also used flow cytometry to estimate the genome size of *C. gynandra*. Nuclei were extracted from *A. thaliana* and *C. gynandra* leaves and run separately and together on a flow cytometer (Fig. 5). Because the 2C genome of *A. thaliana* genome is known to be 135Mb (http://www.arabidopsis.org), and 4C, 8C, and 16C content was also detectable, this allowed us to estimate that the genome of *C. gynandra* is approximately 956Mb (Fig. 5).

## Discussion

### In *A. thaliana*, both M and BS cells undergo endoreduplication

By labelling and isolating nuclei from the BS of *A. thaliana*, we showed directly that endoreduplication can occur within BS cells of C_3_ leaves. Furthermore, when nuclei were isolated from all leaf cell types, we still detected considerable amounts of endoreduplication. Because the BS only represents about 15% of all cells of the leaf (Kinsman and Pyke, 1998), we infer that endoreduplication had also occurred in M cells of *A. thaliana*. During leaf maturation in *A. thaliana*, there were marked alterations in the extent of endoreduplication, for example the number of 2C nuclei declined from 70 to 20% between 5 and 30 d after germination. Over the same time frame, the number of 4C and >8C nuclei increased from 30 and 15% to 55 and 35%, respectively. This large increase in ploidy as leaves of *A. thaliana* mature agrees with previous analysis of whole leaves and appears to correlate negatively with rates of cell division (Beemster *et al.*, 2005). We did detect 4C nuclei or more in very young leaves, which could be due to either significant heterogeneity in the state of cell proliferation or to endoreduplication having occurred in specific cell types very early during leaf maturation (Donnelly *et al.*, 1999).

Because we detected significant endoreduplication in the small BS cells of *A. thaliana* (Kinsman and Pyke, 1998), this implies that an increase in nuclear DNA content is not always linked to larger cells. This conclusion is consistent with previous work indicating that the positive correlation between ploidy levels and cell size is not ubiquitous. For example, increased ploidy in *A. thaliana TRYPTYCHON* (*try*) or *KAKTUS* (*kak*) mutants was not associated with increased trichome cell size (Marks, 1997), and in mutants of cyclin-dependent kinase A1, while cells were enlarged compared with wild type, nuclear DNA content remained stable (Dissmeyer *et al.*, 2009). We also note that, although endoreduplication can be induced by stress (Lee *et al.*, 2009), this was not required for it to be detected in the BS cells of *A. thaliana*.

Our data, combined with previous work, imply that endoreduplication in BS cells is an ancient and conserved characteristic of the angiosperms. As *A. thaliana* is phylogenetically closely related to *C. gynandra*, and their last common ancestor is thought to have diverged around 35 million years ago (Schranz and Mitchell-Olds, 2006), endoreduplication within their BS cells may represent a characteristic present in the last common ancestor of these species. Endoreduplication in BS cells may in fact be even more ancient because it has also been reported in three species of monocotyledons from the Asparagales and the Lilliales (Olszewska *et al.*, 1983). It is possible that endoreduplication within the BS evolved independently within these two lineages of the monocotyledons, and again in the Brassicales within the dicotyledons, but the most parsimonious explanation is that endoreduplication of BS nuclei is the ancestral condition within angiosperms.

While the role that the BS plays in C_3_ plants is not certain (Leegood, 2002), photosynthesis in these cells impacts on whole plant growth and fitness (Janacek *et al.*, 2009), and the cells themselves are implicated in responding to fluctuations in light intensity (Kangasjarvi *et al.*, 2009) as well as in protection against cavitation (Christin *et al.*, 2013; Griffiths *et al.*, 2013). Whether endoreduplication in BS cells impacts on these key functions will be interesting to determine.

### The role of endoreduplication in BS expansion of C_4_ plants

The increase in nuclear DNA content of both BS and M cells during leaf maturation in *A. thaliana* contrasts with the behaviour of nuclei from these cells in C_4_ *C. gynandra*. While it was clear that endoreduplication occurred within BS cells as leaves of *C. gynandra* matured, when nuclei from whole leaves were assessed, we did not detect an increase in DNA content. As the M of C_4_ *C. gynandra* represents a large proportion of the leaf, this indicates that little endoreduplication occurs in M cells of this species. We therefore conclude that, in C_3_ *A. thaliana*, endoreduplication occurs in both M and BS cells, but that in the C_4_ species *C. gynandra*, this behaviour becomes restricted to the BS.

The control of chloroplast number has previously been linked to both endoreduplication (Butterfass, 1989; Ho and Rayburn, 1991) and cell volume (Pyke and Leech, 1987). The fact that we detect significant endoreduplication in the small BS cells of C_3_ *A. thaliana* but also the large BS cells of C_4_ *C. gynandra* suggested that evolution of increased chloroplast number in BS cells of C_4_ species is not likely to be related to enreduplication in these cells but is co-ordinated with cell expansion. We also propose that endoreduplication is not required for expansion, differentiation, and specialization of the BS, a key characteristic of C_4_ plants, but rather that it is a deeply rooted phenotype found within the angiosperms. Our reasoning for this is as follows. First, BS cells of C_3_ *A. thaliana* undergo significant endoreduplication but are small compared with those of C_4_ *C. gynandra*, and also compared with the M cells of C_3_ *A. thaliana*. Secondly, because the large BS cells associated with Kranz anatomy in leaves of *C. gynandra* are present in very young leaves but endoreduplication occurs later during maturation, the two phenomena are unlikely to be linked. Thirdly, while individual BS cells of C_4_ species such as *C. gynandra* (Fig. 1) (Marshall *et al.*, 2007) and C_4_ lineages within the Aizoaceae, Amaranthaceae, and Asteraceae (Muhaidat *et al.*, 2007) are larger than C_3_ relatives, it is also possible to increase the functional volume of the BS by increasing cell numbers rather than cell size. Because the BS surrounds the veins (Muhaidat *et al.*, 2007), if the number of veins within a leaf increases, the consequence is a larger BS compartment. If the BS as a whole is enlarged because of additional cell division, the individual cells within it could remain the same or actually be smaller. In fact, in *F. trinervia*, *A. rosea*, *Z. mays*, and *S. viridis*, we did not detect large differences in the size of M and BS nuclei, and in maize, *F. trinervia*, and *S. viridis*, individual BS cell area was not larger than that of M cells. This is consistent with previous reports that have documented similar M and BS cell sizes in maize (Pengelly *et al.*, 2011), sorghum, *Urochloa panicoides* (von Caemmerer *et al.*, 2007) and *Flaveria bidentis* (Pengelly *et al.*, 2010). Taken together, the data indicate that alterations to the M:BS ratio often occur independently of alterations to individual cell size and that there is not a simple link between endoreduplication and expansion and specialization of BS cells in *C. gynandra*.

## Supplementary data

Supplementary data are available at *JXB* online.

Supplementary Fig. S1. Flow chart of sample preparation and biparametric cytometric analysis to determine BS ploidy levels in *A. thaliana* and *C. gynandra*.

Supplementary Fig. S2. Image of *Cleome gynandra* leaves used for flow cytometry analysis. Numbers represent days after germination.

Supplementary Fig. S3. Representative flow cytometry profile of 18 000 nuclei of *Arabidopsis* leaves 10 days after germination sorted according to DAPI and GFP fluorescence.

Supplementary Data

## Abbreviations:

BS: bundle sheath
CLSM: confocal laser-scanning microscopy
DAPI: 4′-6-diamidino-phenylindole
GFP: green fluorescent protein
M: mesophyll.
