# Supplementary material for: Endoreduplication is not involved in bundle-sheath formation in the C4 species Cleome gynandra
Source: J Exp Bot. 2013 Nov 12;65(13):3557–66. doi: 10.1093/jxb/ert350 (PMC4085951; doi:10.1093/jxb/ert350)
Supplement: Supplementary Data [file supp_65_13_3557__index.html]

Endoreduplication is not involved in bundle-sheath formation in the C4 species Cleome gynandra — Endoreduplication is not involved in bundle-sheath formation in the C4 species Cleome gynandra — Endoreduplication is not involved in bundle-sheath formation in the C4 species Cleome gynandra — Supplementary Data 

# Endoreduplication is not involved in bundle-sheath formation in the C4 species *Cleome gynandra*

## Supplementary Data

Data files

**Files in this Data Supplement:**

- Supplementary Data - Supplementary Data
