# Supplementary material for: Endoreduplication is not involved in bundle-sheath formation in the C4 species Cleome gynandra
Source: J Exp Bot. 2013 Nov 12;65(13):3557–66. doi: 10.1093/jxb/ert350 (PMC4085951; doi:10.1093/jxb/ert350)
Supplement: Supplementary Data [file supp_ert350_jexbot107771_file001.pdf]

**Endoreduplication of Bundle Sheath cells in the $C_4$ species *Cleome gynandra* and the $C_3$ model *Arabidopsis thaliana***

*Julian Hibberd, Sylvain Aubry, and Jana Knerova*

**Supplementary Figure S1.**

Flow chart of sample preparation and biparametric cytometric analysis to determine BS ploidy levels in *A. thaliana* and *C. gynandra*. (A) Samples were chopped and prepared using the Galbraith method (Zhang *et al.*, 2005). (A) After appropriate gating of the nuclei signals, DAPI signals were plotted against GFP signals to isolate GFP+ nuclei from BS cells. The inner square shows GFP+ nuclei used for ploidy quantification. Arrows show 2C, 4C and 8C DNA nuclear contents.

**Supplementary Figure S2.**

Image of *Cleome gynandra* leaves used for flow cytometry analysis. Numbers represent days after germination.

**Supplementary Figure S3.**

Representative flow cytometry profile of 18,000 nuclei of Arabidopsis leaves 10 dag sorted according to DAPI (vertical axis) and GFP (horizontal axis) fluorescence. Similar gating was used to generate Fig 3D & F.

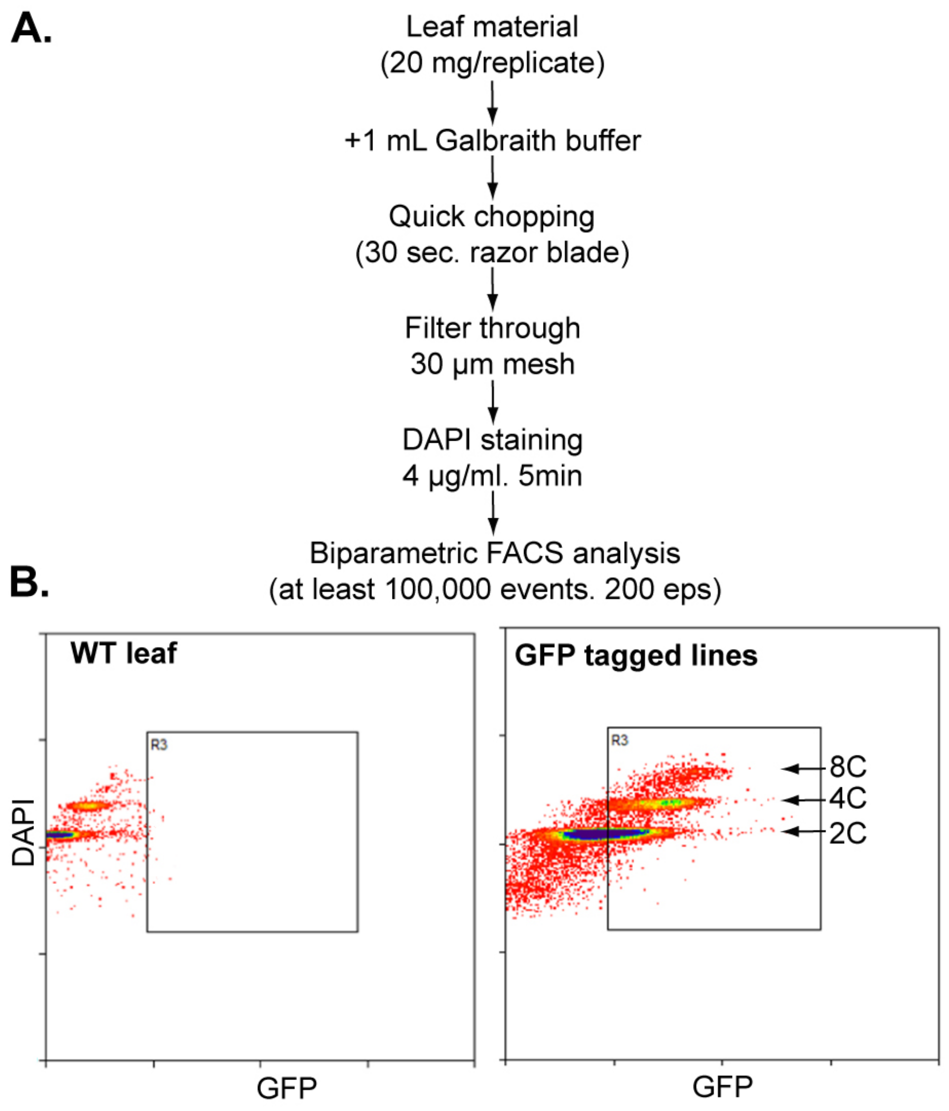


Supplementary Figure 1

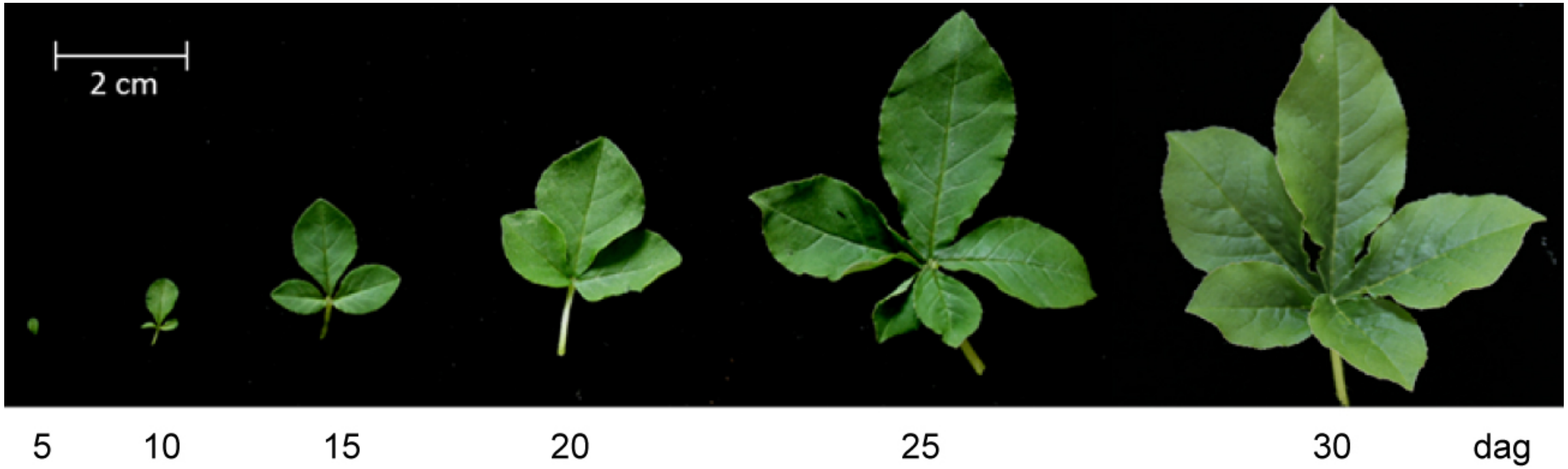


**Supplementary Figure 2**

Biparametric flow cytometry profile of Arabidopsis leaf
10 dag. (18,000 nuclei)

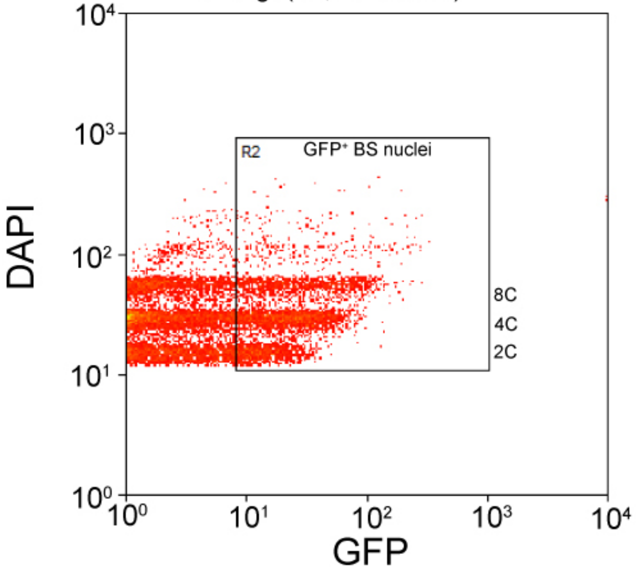


**Supplementary Figure 3**
